# Surface PEGylated Cancer Cell Membrane-Coated Nanoparticles for Codelivery of Curcumin and Doxorubicin for the Treatment of Multidrug Resistant Esophageal Carcinoma

**DOI:** 10.3389/fcell.2021.688070

**Published:** 2021-07-27

**Authors:** Yi Gao, Yue Zhu, Xiaopeng Xu, Fangjun Wang, Weidong Shen, Xia Leng, Jiyi Zhao, Bingtuan Liu, Yangyun Wang, Pengfei Liu

**Affiliations:** ^1^Department of Gastroenterology, The Affiliated Jiangyin Hospital of Xuzhou Medical University, Jiangyin, China; ^2^State Key Laboratory of Radiation Medicine and Protection, Medical College of Soochow University, Suzhou, China

**Keywords:** esophageal carcinoma, multidrug resistant, homologous targeting, doxorubicin, curcumin

## Abstract

**Objective:**

The emergence of multi-drug resistance (MDR) in esophageal carcinoma has severely affected the effect of chemotherapy and shortened the survival of patients. To this end, we intend to develop a biomimetic nano-targeting drug modified by cancer cell membrane, and investigate its therapeutic effect.

**Methods:**

The degradable poly(lactic-co-glycolic acid) (PLGA) nanoparticles (NPs) co-loaded with doxorubicin (DOX) and curcumin (Cur) were prepared by solvent evaporation method. TE10 cell membrane and Distearoyl phosphatidylethanolamine-polyethylene glycol (DSPE-PEG) were then coated on the PLGA NPs by membrane extrusion to prepare the PEG-TE10@PLGA@DOX-Cur NPs (PMPNs). Size and zeta potential of the PMPNs were analyzed by lazer particle analyzer, and the morphology of PMPNs was observed by transmission electron microscope. The TE10 cell membrane protein on PMPNs was analyzed by gel electrophoresis. The DOX-resistant esophageal cancer cell model TE10/DOX was established through high-dose induction. The *In vitro* homologous targeting ability of PMPNs was evaluated by cell uptake assay, and the *in vitro* anti-tumor effect of PMPNs was assessed through CCK-8, clone formation and flow cytometry. A Balb/c mouse model of TE10/DOX xenograft was constructed to evaluate the anti-tumor effect *in vivo* and the bio-safety of PMPNs.

**Results:**

The prepared cell membrane coated PMPNs had a regular spherical structure with an average diameter of 177 nm. PMPNs could directly target TE10 and TE10/DOX cells or TE10/DOX xenografted tumor and effectively inhibit the growth of DOX-resistant esophageal carcinoma. Besides, the PMPNs was confirmed to have high biosafety.

**Conclusion:**

In this study, a targeted biomimetic nano-drug delivery system PMPNs was successfully prepared, which overcome the MDR of esophageal carcinoma by co-delivering DOX and sensitizer curcumin.

## Introduction

Esophageal carcinoma (ESCA) is a common tumor in the digestive system (Alsop and Sharma, 2016). It is insidious, almost asymptomatic in the early stage, and most patients have entered the advanced stage when discovered, which significantly increases the difficulty of treatment and the chance of recurrence (Domper Arnal et al., 2015; Ohashi et al., 2015). In addition to surgical resection, two strategies have been employed to improve the therapeutic effect of ESCA, one is to seek more effective drugs, such as molecular-targeted drugs; the other one is drug combination, through combining one chemotherapy drug with other chemotherapy drugs, molecular-targeted drugs or sensitizers, which assemble different mechanisms to enhance the effect of chemotherapy (Hu et al., 2020; Zhao et al., 2020). However, the MDR of esophageal carcinoma is still a difficult problem to overcome. Therefore, the combination of chemotherapeutics and sensitizers has received widespread attention. DOX is the first-line clinical drug for the treatment of esophageal carcinoma, but its narrow therapeutic window, cardiotoxicity, and other side effects and drug resistance restrict its application (Amjadi et al., 2019). Studies have shown that the combined use of DOX and sensitizers can significantly enhance its antitumor ability (Oh et al., 2015). Cur, an extract of traditional Chinese medicine, was found to be a potential chemotherapeutic sensitizer, which can reverse MDR and reduce the side effects of chemotherapeutic drugs, to achieve the effect of synergistic treatment of tumor (Xu et al., 2020).

In recent years, with the development of nanotechnology, tumor targeting therapy mediated by nanoscale materials has aroused widespread attention. Lee et al. (2020) synthesized PLGA NPs loaded with DOX. Through the interaction between DOX and sialic acid on tumor cells, the targeting capacity of NPs and the permeability of the tumor were simultaneously enhanced, which was beneficial for NPs enter the tumor to exert their efficacy. Drug-loaded nano-system has many advantages. On the one hand, it enhances the solubility and bioavailability of drugs; on the other hand, it has the sustained effect for drug release, which makes the drug concentration in blood stable in a safe and effective range over a period of time, thus extending the efficacy time and reducing the toxicity and side effects of drugs (Li et al., 2017; Ma et al., 2013; Prakash and Dhesingh, 2017; Son and Kim, 2017). The frequently used drug delivery nano-system include liposomes, solid lipid nanoparticles, polymer nanoparticles, dendrimers, nanoemulsions, inorganic porous silicon nanoparticles, magnetic iron oxide nanoparticles, etc. (Rabanel et al., 2012; Carita et al., 2018). PLGA is a biodegradable functional polymer organic compound with good biocompatibility, spheronization, and non-toxicity. It has been widely used in drug delivery and medical materials in recent years (Han et al., 2016; Lagreca et al., 2020).

In the multiple researches of nano-therapeutics, direct targeting of tumors by surface modification of NPS has always been a hot topic. It has been reported that NPs can be enriched in tumor tissues by passive targeting, that is, taking advantage of the enhanced permeability and retention (EPR) effect (Maeda, 2017). However, the enrichment efficiency of EPR effect is not high, exhibiting obvious diversity and heterogeneity (Danhier, 2016). Therefore, researchers have gradually turned their attention to how to achieve the active targeting function of NPs. The emergence of the cell membrane wrapping technology provides a new modification and camouflage strategy for the development of NPs with high targeting and low immunogenicity. The first report of this technology was Hu et al. (2011), they wrapped NPs with erythrocyte membrane to allow it to evade immune surveillance. Different cell membrane coating endows NPs different functions. For example, NPs wrapped with erythrocyte membrane can evade immune surveillance, NPs wrapped with stem cell membranes show good targeting anti-tumor effects, and macrophage membranes wrapped NPs can reduce the effects of opsonin and prolong circulation time (Wang et al., 2018). Compared to wrapping with other cell membranes, NPs encapsulated with tumor cell membranes have excellent targeting effect (Zhen et al., 2019). In addition to accumulate to the target sites by EPR effect, it can also actively aggregate to tumor sites through homologous recognition mechanism. Research by Fang et al. (2014) showed that the NPs wrapped with tumor cell membrane were 40 and 20 times more efficient to be taken up by tumor cells than erythrocyte membrane-coated NPs and the naked NPs, respectively. Hence, the use of tumor cell membranes to encapsulate drug-loaded NPs is an effective tool to achieve efficient drug delivery and targeted attack on tumors. In addition, NPs are unstable in the body and are easily swallowed by the reticuloendothelial system (RES), resulting in shorter circulation time in the body and reduced drug release, thereby reducing clinical effects. The most common solution to solve this problem is to modify the surface of NPs with PEG. PEG is a non-toxic, non-immunogenic and antigenic, highly water-soluble compound, and has no effect on the release of drugs. PEGylation can enhance the stability of the NPs, prevent the binding of NPs and plasma proteins, thereby reducing the clearance rate of the reticuloendothelial system and prolonging the half-life of the drug, besides, it can reduce the immunogenicity of NPs and enhance drug retention (Yadav and Dewangan, 2021).

Herein, we designed and synthesized PMPNs for targeted therapy of MDR esophageal carcinoma. The PLGA NPs co-loaded anti-cancer drugs DOX and Cur was prepared by solvent volatilization method, thus enhanced the anti-tumor effect through the synergistic effect of the drugs. Then, the extracted TE10 cell membrane and DSPE-PEG were self-assembled to wrap the drug-loaded PLGA NPs, so that the homologous recognition mechanism between tumor cells could be used to achieve tumor targeting. The PMPNs could avoid the rapid clearance of the RES to a certain extent and increase the circulation time in the body. It was confirmed that PMPNs have a good therapeutic effect on MDR esophageal carcinoma, it could not only overcome the side effects of chemotherapy drugs, but also target tumor sites and increase the residence time of drugs in tumor. Our research will become an innovative strategy for treating MDR esophageal carcinoma.

## Materials and Methods

### Materials

Cell lines were all obtained from American Type Culture Collection(ATCC). DMEM medium, Fetal Bovine Serum (FBS) were purchased from Hyclone (United States). Penicillin and Streptomycin mixture, trypsin ethylenediaminetetraacetic acid (EDTA), Coomassie Brilliant Blue Dye, Bull Serum Albumin (BSA), ECL luminescence reagent, 4′6-diamidino-2-phenylindole (DAPI) reagent, Propidium Iodide (PI) reagent, Cell Counting kit (CCK) - 8, Hematoxylin-Eosin (HE) staining kit, membrane protein extraction kit and TdT-mediated dUTP Nick-End Labeling (TUNEL) apoptosis assay kit were obtained from Sangon Biotech (China). The malondialdehyde (MDA) content detection kit and superoxide dismutase (SOD) activity detection kit was purchased from Solarbio life sciences (China), the Glutathione peroxidase (GSH-Px) detection kit was purchased from Leagene Biotechnology (China). PLGA (Lactic acid: Glycolic acid = 50:50), Polyvinyl alcohol (PVA), DSPE-PEG and tetramethyl rhodamine (TRITC) were purchased from Sigma-Aldrich (United States). DOX and Cur was provided by Meilunbio (China). The polycarbonate membrane (220 nm), polyvinylidene fluoride (PVDF) membrane and Transwell chamber were purchased from Millipore (Germany). The monoclonal antibody against ABCB1 (ab231535), ABCC1 (ab69296), LGR5 (ab75850), CD44 (ab51037), Bax (ab270742), Cyto-C (ab133504), cleaved caspase 3 (ab214430) and β-actin (ab179467) were purchased from Abcam (United Kingdom). The secondary antibodies including Goat anti-mouse IgG (ab205719) and Goat anti-rabbit IgG (ab205718) were obtained from Thermo Fisher Scientific (United States).

### Cell Culture

TE10, L02, and A549 cells were used in this study. These cells were first resuscitated with Dulbecco’s Modified Eagle Medium (DMEM) complete medium supplemented with 10% fatal bovine serun (FBS), 100 U/mL penicillin and streptomycin, then cultured and passaged in an incubator containing 5% CO_2_ at 37°C.

### Cell Membrane Extraction

The digested TE10 cells (1 × 10^8^) were collected and centrifuged at 700 g for 5 min. The cells were suspended in pre-cooled phosphate buffer saline (PBS) buffer (pH = 7.4) and then centrifuged at 700 g for 5 min. According to the instruction of Cell membrane protein and cytoplasmic protein extraction kit (Beyotime, China), specifically, TE10 cells were collected and washed 3 times with pre-cooled PBS, and then resuspended with 1 mL reagent A. After ice bathing for 15 min, the cell suspension was placed in liquid nitrogen and room temperature (RT) successively, and then freeze-thaw for twice. After that, it was centrifuged at 700 g, 4°C for 10 min, then the supernatant was centrifuged 14,000 g, 4°C for 30 min. Finally, the collected cell pellets were suspended with membrane protein extraction reagent containing Phenylmethylsulfonyl fluoride (PMSF). The cells were ice-bathed in this hypotonic lysis buffer for 10–15 min and then lysed with ultrasound. Cell lysate was centrifuged at 700 g for 10 min and the supernatant was then centrifuged at 14,000 g for 30 min. The precipitate was the extracted cell membrane.

### Preparation of Drug-Loaded PLGA NPs

**SCHEME 1:**
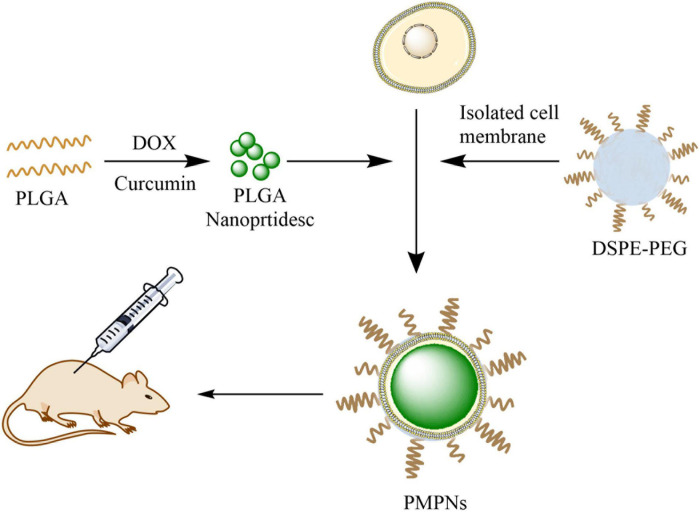
Illustration of the preparation of PMPNs and *in vivo* therapy. PLGA was first dual-loaded with DOX and Cur to form PLGA NPs, then the isolated TE10 cell membrane and DSPE-PEG were added and self-assembled on PLGA NPs to form PMPNs. The biomimetic nano-drug PMPNs were applied for *in vivo* therapy of multidrug resistant Esophageal Carcinoma.

To prepare the dual-loaded PLGA NPs, we first dissolved 10 mg Cur and 300 mg PLGA (50:50) in 10 mL of dichloromethane under sonication to prepare the oil phase. Next, 10 mg DOX was dissolved in 1 mL ddH_2_O as water phase. The oil and water phase were mixed up with ultrasonic wave (Kunshan KQ-250E Ultrasonic Instrument, China) for 10 min. Then, the mixed emulsion was quickly added to 100 mL PVA (2%) solution under high-speed magnetic stirring to form microspheres. Finally, the microspheres were stirred magnetically at 800 rpm for 8 h in RT to completely evaporate methylene chloride. The sample was frozen for 12 h after washing with ddH_2_O for three times and then vacuum dried (Christ Freeze dryer, Germany) for subsequent use (Scheme 1).

### Preparation of PMPNs

The TE10 cell membrane and DSPE-PEG15000 at a 5 mg/mL concentration were blended with drug-loaded PLGA NPs under ultrasonic. Then, Avanti Mini-Extruder (Beckman Coulter, United States) was used to squeeze 10 times on a 200 nm polycarbonate porous membrane, and then the solution was centrifuged to remove excess cell membrane (Scheme 1).

### Characterization of PMPNs

After preparing the appropriate concentration of PLGA NPs, TE10 vesicles, TE10-PLGA and PEG-TE10-PLGA NPs, the size and zeta potential were measured with laser particle size analyzer (Malvern, United Kingdom). Then, we dropped 10 μL of them on a 200-mesh copper mesh with carbon-supported film, after keeping for 5 min at RT, the excess liquid was absorbed with filter paper. After that, 5 μL of 1% phosphotungstic acid was dropped on the copper mesh and dyed for 2 min, then the structure of the samples was observed with a transmission electron microscope (FEI Talos L120C, United States).

To characterize the cancer cell membrane proteins on the surface of the NPs, we mixed PLGA NPs, TE10 vesicles, TE10@PLGA, and PEG-TE10-PLGA NPs with the loading buffer, and denatured at 95°C for 5 min. Then, samples and the protein marker were slowly added to the wells of two gels and underwent electrophoresis at constant voltage of 100 V (Bio-rad Mini-ProteanTetra, United States). After electrophoresis, the gels were taken out and one gel was stained with Coomassie Brilliant Blue Solution and photographed for analysis. Another gel was performed trans-membrane assay. After the experiment, the membrane was blocked with 1% BSA, then incubated with the primary antibody LGR5 and CD44 (1: 2,000 dilution) at 4°C overnight, followed by secondary antibody (1: 10,000 dilution) at 37°C for 1 h. Finally, the membrane was rinsed with PBST and developed with ECL.

To determine the effect of surface modification of PEG with different molecular weights on the properties of NPs, BSA adsorption experiment was first performed, briefly, PLGA NPs coated with PEG of different molecular weight (MW = 5,000, 10,000, 15,000) were prepared, and then incubated with TRITC-labeled BSA for 10, 30, and 60 min, respectively. After rinsed with PBS, the BSA adsorption capacity of PEGylated NPs was evaluated via the fluorescence intensity of TRITC measured by Fluorescence spectrophotometer (PerkinElmer FL8500, South Korea). Then the uptake of different molecular weight PEG-modified NPs by macrophage was evaluated by flow cytometry (BD FACSCalibur, United States) and Laser scanning confocal microscope (LSCM) (Olympus FV3000, Japan).

### Establishment of the Drug-Resistant Cell Line TE10/DOX

High-dose intermittent induction method was used to establish DOX-resistant TE10 cell line (Xiao et al., 2017). ① TE10 cells in the logarithmic growth phase were inoculated in 96-well plates. After 24 h, the cells were treated with DOX at the final concentrations of 0.032, 0.16, 0.8, 4, and 20 μM, and continued to culture for 48 h. Then, CCK-8 method was applied to detect the *IC*_50_ value. ② TE10 cells were inoculated in a 6 cm petri dish and grew to logarithmic growth phase, then DOX with a final concentration of 100 μM was added and incubated for 12 h. After that, the drug-containing medium was discarded and the cells were cultured in DOX-free medium for several generations. After the cells grew well, the above method was repeated until the cells could be cultured in 100 μM DOX for a long time, thus the drug-resistant human esophageal cancer cell model TE10/DOX was established.

Identification of the prepared DOX-resistant cells: First, the *IC*_50_ values of TE10 cells and TE10/DOX cells were detected by CCK-8 assay. Next, the expression of resistance proteins ABCB1 and ABCC1 in TE10 cells and TE10/DOX cells was detected by Western blot (WB). The primary antibody anti-ABCB1 was 1:5,000 diluted, anti-ABCC1 was 1:3,000 diluted, and the secondary antibody goat anti-rabbit IgG and goat anti-mouse IgG were 1:10,000 diluted.

### *In vitro* Drug Release

Low-speed centrifugation was used to detect the *in vitro* drug release of PMPNs. PLGA@DOX, TE10-PLGA@DOX, and PEG-TE10-PLGA@DOX with equal DOX concentration were placed in a centrifuge tube, respectively, and then shaked at 37°C at 100 rpm. Then the tubes were taken out at the preset time point and centrifuged at 1,000 rpm for 5 min. The supernatant was collected and measured at OD_480__nm_, the unloaded NPs under the same conditions were used as the blank reference.

### Cell Uptake Experiments *in vitro*

To evaluate whether the prepared NPs could be uptaken by tumor cells *in vitro*, TE10 cells were first seeded in a 24-well plate containing cell slides. After the cells were completely attached, PLGA@DOX, TE10-PLGA@DOX, and PEG-TE10-PLGA@DOX were added and co-incubated for 4 h. Then, the culture medium was removed, the cells were washed with PBS for three times and then fixed with 4% paraformaldehyde for 15 min. At last, the slides were pasted onto the glass slide with DAPI-containing mounting reagent, then observed and recorded under the LSCM.

Next, flow cytometry was used to analyze the uptake of NPs quantitatively: Firstly, cell culture was conducted according to the above steps and treated with NPs for 4 h. After rinsing with PBS, cells were collected and quantitatively analyzed via flow cytometry.

### Tumor Targeting Experiments *in vitro*

L02, A549, TE10, and TE10/DOX cells were used to determine the tumor targeting function. Cells were incubated with PMPNs. Other operations were carried out as described above.

### Determination of Anti-tumor Ability *in vitro*

CCK-8 assay, clone formation experiment and AnnexinV-PI apoptosis assay were used to investigate the *in vitro* anti-tumor activity of the PMPNs.

CCK-8 assay: TE10/DOX cells were inoculated in a 96-well plate with 200 μL per well (about 2 × 10^4^ cells). Cells were divided into three groups: PLGA@Cur + DOX, TE10-PLGA@Cur + DOX and PEG-TE10-PLGA@Cur + DOX groups, different concentrations of NPs were added to each group of cells, and the concentrations of loading drug were 3.125, 6.25, 12.5, 25, and 50 μg/mL, respectively. After incubation for 24 h, 10 μL of CCK-8 reagent was added to each well, and incubated for another 2 h. The OD_450__nm_ was measured by microplate reader (Bio-Tek Epoch, United States).

Clone formation experiment: TE10/DOX cells were first inoculated in a 6-well plate, then the cells were divided into four groups: PBS, PLGA@Cur + DOX, TE10-PLGA@Cur + DOX and PEG-TE10-PLGA@Cur + DOX. After treatment for 24 h, cells were digested into single cells, then inoculated in a dish at a density of 200 cells/well, and continue cultured for 1–2 weeks. After that, the supernatant was discarded and washed twice with PBS. After immobilization, cells were stained with Giemsa staining solution for 30 min. The number of clones was counted with a microscope.

In addition, flow cytometry was used to detect the apoptotic proportions, and WB method was performed to assess the expression of apoptosis-related proteins. The detected cytochrome C (Cyto-C) is the content of Cyto-C released by mitochondria into the cytoplasm. Briefly, after the cells were lysed, the lysate was centrifuged twice at 4°C for 10 min, and the obtained supernatant was centrifuged at 4°C, 10,000g for 30 min, then the supernatant was centrifuged at 4°C, 100,000 g for 1 h to precipitate organelles including mitochondria, and the final obtained supernatant was used for WB detection. The experiment was performed as described above. Primary antibodies were diluted as follow: anti-Bax (1:1,000), anti-Cyto-C (1:2,000), anti-cleaved cas 3 (1:2,000), and anti-β-actin (1:5,000). The secondary antibodies were all diluted at 1:1,000.

To study the drug synergy of DOX and Cur, we prepared TE10-PLGA@Cur, TE10-PLGA@DOX and TE10-PLGA@Cur + DOX, respectively, and incubated them with TE10/DOX cells. CCK-8 assay, flow cytometry and WB were performed as described above to assess cell viability and cell apoptosis. Then, transwell assay was conducted to assess cell invasion. In brief, cells treated by different NPs were seeded into the upper transwell chamber, after 24 h, cells in lower chamber were immobilized with aldehyde fixative for 30 min and stained with 0.1% crystal violet for 20 min, the results were recorded with the microscope.

### Biodistribution of PMPNs *in vivo*

We chose 6–8 weeks old female Balb/c nude mice as animal model. The animal experiment had been approved by the Ethics Committee of Jiangyin People’s Hospital, the Jiangyin Clinical College of Xuzhou Medical University. To investigate the biodistribution of PEG-TE10-PLGA NPs *in vivo*, we injected 100 μL PBS containing 5 × 10^6^ TE10/DOX cells into the subcutaneous breast. When the tumor grew to nearly 300 mm^3^, PLGA@DiR, TE10-PLGA@DiR, and PEG-TE10-PLGA@DIR (5 mg/kg) were injected through the tail vein, and the control group was injected with equal volume of saline. After 0, 8, 24, and 48 h, tail vein blood (200 μL) was taken and the serum was collected by centrifugation. The drug concentration in blood was evaluated through the fluorescence intensity of DiR. The fluorescence intensity at the tumor site was measured with a live animal imager (Perkin Elmer PE IVIS SPECTRUM, United States). After 48 h of intravenous injection, the mice were dissected, heart, liver, spleen, lung, kidney and tumor were taken out and the fluorescence intensity in each organ and tumor tissue was measured.

### Determination of the Anti-tumor Ability and Biological Safety *in vivo*

The mice were first inoculated with TE10/DOX cells, when the tumors grew to about 100 mm^3^, the mice were randomly divided into four groups: saline, PLGA@Cur + DOX, TE10-PLGA@Cur + DOX and PEG-TE10-PLGA@Cur + DOX, with 5 mice in each group. Then we injected Cur/DOX into the tail vein at a dose of 5 mg/kg or corresponding volume of saline. The drug was administered every 3 days, and the tumor volume and body weight were measured at the same time. On the day 16, the mice were sacrificed, then the tumor tissues were stripped and weighed (Scheme 1).

Next, the obtained tumor tissues were soaked in formalin solution, then embed in paraffin and sliced. HE staining kit and the TUNEL apoptosis detection kit were applied to detect the degree of tumor necrosis and apoptosis. For HE staining, paraffin sections were first dewaxed with xylene and hydrated with low to high concentrations of alcohol, then stained with hematoxylin and eosin successively. After washing off the excess dye with water, the slices were dehydrated using graded alcohol and vitrification by xylene. Finally, the slices were sealed with resin and observed under microscope. As for TUNEL method to detect apoptosis, the samples were first deparaffinized and hydrated, then adding the proteinase K for permeabilization. After rinsing with PBS, adding TdT enzyme working solution for labeling. Finally, observing the green fluorescence with the LSCM. At the same time, the heart, liver, spleen, lung and kidney were sectioned and the damage degree of the main organs was evaluated by HE staining.

For the purpose of evaluating the effect of different surface-modified NPs on the oxidative stress level of mouse cardiomyocytes, we cut off mouse myocardium and rinsed with pre-cooled PBS. Then the homogenate was prepared and centrifuged at 2,500 rpm at 4°C for 10 min. The content of MDA, GSH-Px, and SOD in cardiomyocytes was detected by kit. Ultraviolet spectrophotometry was applied to detect the content of MDA, GSH-Px, and SOD in mouse cardiomyocytes. The prepared tissue homogenate was mixed with working reagents in the kit, and then the concentration was detected and calculated under the corresponding absorbance.

In addition, the blood was collected for biochemical analysis (Cobas c311, United States) to detect the concentration of alanine aminotransferase (ALT), aspartate aminotransferase (AST), white blood cells (WBC), red blood cells (RBC) and platelets (PLT) in serum.

Moreover, to evaluate the survival of mice in each group, we took the same number of mice, repeated tumor transplantation and drug treatment according to the above operation, and administered the drug every 3 days. The mice of each group were fed until the 40th day, and the survival time of each group was recorded.

### Statistical Analysis

Three parallel experiments were performed for each experiment, and the results were expressed as mean ± standard deviation. One-way analysis of variance was used to compare the differences between the two sets of data. *P* < 0.05 was considered statistically significant.

## Results

### Preparation and Characterization of PMPNs

**FIGURE 1:**
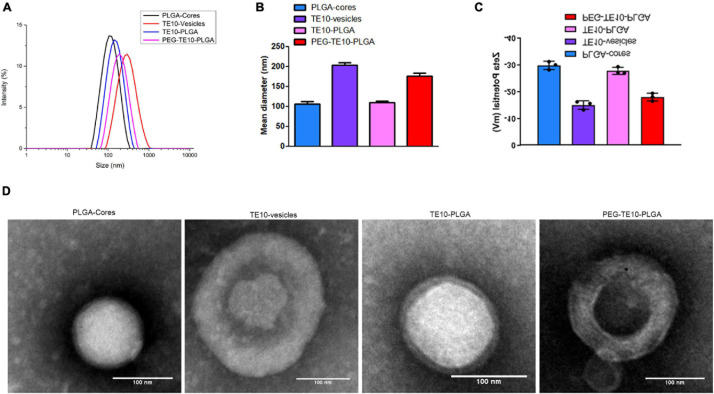
Characterization of PMPNs. Malvern laser particle size analyzer was used to analyze the particle size distribution **(A)**, the average diameter **(B)**, and the Zeta potential **(C)** of PLGA-Cores, TE10-vesicles, TE10-PLGA and PEG-TE10-PLGA. **(D)** TEM imaging of the four PMPNs. Scale bar = 100 nm.

In this research, we first prepared PLGA NPs loaded with anti-tumor drugs DOX and Cur. It was shown in Figures 1A–C that the hydrodynamic diameter of the PLGA core was about 106 nm and the hydrodynamic diameter of the extracted TE10 cell membrane was about 204 nm. We mixed the extracted TE10 cell membrane with the PLGA core to prepare TE10-PLGA NPs, and the hydrodynamic diameter increased to 109 nm. These indicated that TE10 cell membrane had been successfully coated on the surface of PLGA core. Next, we mixed the PLGA core with equal concentration of the extracted TE10 cell membrane and DSPE-PEG, it was found that the hydrodynamic diameter of the obtained PEG-TE10-PLGA NPs increased to 177 nm, indicating that PEG was also successfully inserted into the surface of the NPs. Here, we evaluated the effect of PEG modification with different molecular weights on NPs. DSPE-PEG5000, DSPE-PEG10000 and DSPE-PEG15000 were modified on the surface of PLGA NPs, respectively, it was confirmed that DSPE-PEG15000 has the least adsorption of BSA, which allowing NPs to escape macrophages’ uptake (Supplementary Figure 1). Transmission Electron Microscopy (TEM) images in Figure 1D proved that both of TE10-PLGA and PEG-TE10-PLGA displayed a core-shell structure and the hydrodynamic diameter of the NPs was consistent with the Dynamic Light Scattering (DLS) results.

### Homologous Targeting of PMPNs to Tumor Cells *in vitro*

**FIGURE 2:**
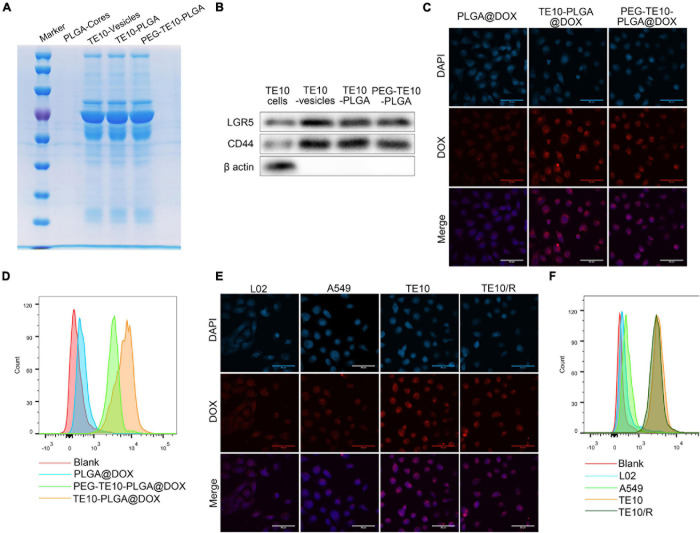
Specific uptake of PMPNs by homologous tumor cells. **(A)** SDS-PAGE analysis of PLGA-Cores, TE10-vesicles, TE10-PLGA and PEG-TE10-PLGA. **(B)** Western blot analysis of biomarkers of TE10 cell membrane on PLGA-Cores, TE10-vesicles, TE10-PLGA and PEG-TE10-PLGA. **(C,D)** Evaluating the uptake of PLGA@DOX, TE10-PLGA@DOX, and PEG-TE10-PLGA@DOX into TE10 cells by laser confocal microscopy **(C)** and flow cytometry **(D)**. **(E,F)** L02, A549 and TE10/DOX cells were used to evaluate the tumor targeting capacity of PEG-TE10-PLGA@DOX by laser confocal microscopy **(E)** and flow cytometry **(F)**.

First, SDS-PAGE electrophoresis assay and WB assay were used to further verify whether TE10 cell membrane was successfully encapsulated on PLGA NPs. The results were shown in Figures 2A,B. Uncoated NPs (PLGA-core) had no protein bands and specific surface markers (LGR5 and CD44) of TE10 cells, while NPs coated with TE10 membrane (TE10-PLGA and PEG-TE10-PLGA) had protein bands similar to TE10 vesicles, and the protein markers were also possessed. These results certified that the cell membrane had been successfully coated on the surface of the NPs, and the membrane protein was well retained during the preparation process.

Next, in order to verify the homologous targeting effect of the PMPNs, we used LSCM and flow cytometry to detect the uptake of PLGA@DOX, TE10-PLGA@DOX and PEG-TE10-PLGA@DOX NPs by TE10 cells and the intracellular drug distribution. As shown in Figures 2C,D, the fluorescence intensity of the TE10 cell membrane-coated NPs in TE10 cells was significantly higher than that of the uncoated PLGA@DOX NPs.

In addition, we established and identified DOX-resistant TE10 cell model, as shown in Supplementary Figure 2, the *IC*_50_ of TE10 cells was 12.61 μM, while the *IC*_50_ of TE10/DOX celsl was 45.01 μM. Besides, the resistance proteins ABCB1 and ABCC1 were found high-expressed in TE10/DOX cells. Then we evaluated the targeting specificity of PEG-TE10-PLGA@DOX NPs to different cells (L02, A549, TE10 and TE10/DOX cells). It was found that the fluorescence intensity in TE10 and TE10/DOX cells was significantly higher than that in A549 and L02 cells (Figures 2E,F).

### Evaluation of the *in vitro* Anti-tumor Activity

**FIGURE 3:**
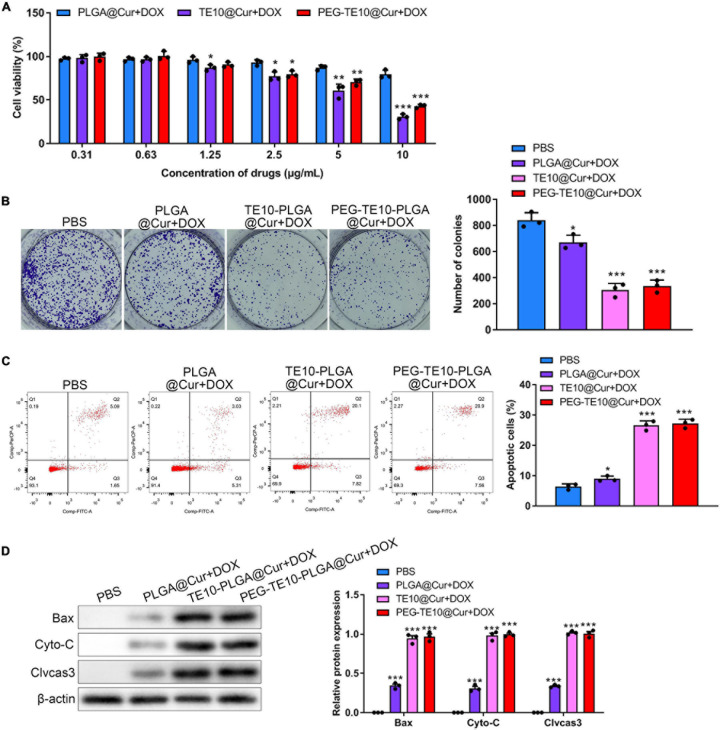
*In vitro* anti-tumor activity of PMPNs. **(A,B)** Effect of PLGA@Cur + DOX, TE10-PLGA@Cur + DOX, and PEG-TE10-PLGA@Cur + DOX on proliferation of TE10/DOX cells was assessed through **(A)** CCK-8. **(B)** Clone formation assay **(C)** flow cytometry analysis of TE10/DOX cell apoptosis induced by PLGA@Cur + DOX, TE10-PLGA@Cur + DOX, and PEG-TE10-PLGA@Cur + DOX. **(D)** Western blot analysis of expression of apoptosis-related protein in TE10/DOX cells after treated with different NPs. *P < 0.05, **P < 0.01, ***P < 0.001 vs. control group.

In order to evaluate the anti-tumor effect of the synthesized NPs *in vitro*, CCK-8 assay and clone formation assay were performed to evaluate the toxic effects of PLGA@Cur + DOX, TE10@Cur + DOX and PEG-TE10-PLGA@Cur + DOX on TE10/DOX cells. We found that when the drug concentration was over 25 μg/mL, TE10-PLGA@Cur + DOX and PEG-TE10-PLGA@Cur + DOX had significantly enhanced toxic effects on TE10/DOX cells, while PLGA@Cur + DOX had no obvious toxic effects (Figure 3A). In the clone formation assay, the number of clones in the cells treated with TE10-PLGA@Cur + DOX and PEG-TE10-PLGA@Cur + DOX was significantly less than that treated with PBS and PLGA@Cur + DOX (Figure 3B). The above results indicated that the drug carrier PLGA NPs had good biocompatibility and could well controlled the drug release.

After that, we further investigated the *in vitro* anti-tumor effect of PLGA@Cur + DOX, TE10-PLGA@Cur + DOX and PEG-TE10-PLGA@Cur + DOX using apoptosis analysis. As shown in Figure 3C, TE10-PLGA@Cur + DOX treated cells had the highest apoptosis ratio, while the apoptosis ratio of PBS and PLGA@Cur + DOX treated cells was much lower. Several apoptosis-related proteins were detected by WB and the expression of the Cyto-C released into cytoplasm, Bax and Cleaved caspase 3 in the cells treated with TE10-PLGA@Cur + DOX and PEG-TE10-PLGA@Cur + DOX was significantly higher than that in the other two groups (Figure 3D). This result confirmed that the combination of Cur and DOX had a strong anti-tumor effect *in vitro*, and the encapsulation of PLGA effectively avoided rapid leakage of the drug. Furthermore, we confirmed that there was a synergistic anti-tumor effect between Cur and DOX through CCK-8 assay, apoptosis analysis, and transwell assay. The anti-tumor effect of combination DOX and Cur was much better than DOX or Cur alone (Supplementary Figure 3).

### Biodistribution of PMPNs *in vivo*

**FIGURE 4:**
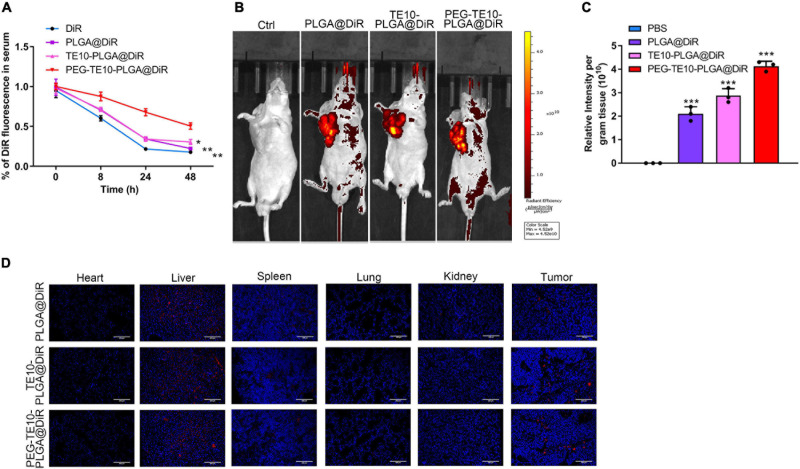
Pharmacokinetics and biodistribution of PMPNs. **(A)** Changes of DiR fluorescence in serum of mice after treatment with DiR, PLGA@DiR, TE10-PLGA@DiR and PEG-TE10-PLGA@DiR. *In vivo* fluorescence imaging **(B)** and quantification of mice **(C)**. **(D)** Biodistribution of PLGA@DiR, TE10-PLGA@DiR and PEG-TE10-PLGA@DiR in main organ and tumor of mice. DiR fluorescence intensity was then quantified. ***P < 0.001 vs. control group.

By means of DiR, a cell membrane probe, we successfully monitored the drug metabolism and biodistribution of the prepared NPs in mice. DiR, PLGA@DiR, TE10-PLGA@DiR and PEG-TE10-PLGA@DiR were injected into the tail vein of nude mice, and the fluorescence intensity of DiR in serum was detected at 0, 8, 24, and 48 h to evaluate the metabolism of NPs in mice. Mice in the PEG-TE10-PLGA@DIR group maintained a higher blood drug concentration 48h after injection (Figure 4A). Supplementary Figure 4 showed the *in vitro* drug release curves of three NPs, PEG-TE10-PLGA@DOX had the slowest drug release. Using the small animal live imaging device, we clearly observed the fluorescence of the xenografted tumor of each mouse and quantitatively analyzed the intensity (Figures 4B,C). In addition, the fluorescence imaging and quantitative analysis of mouse heart, liver, spleen, lung, kidney and tumor tissues showed that the fluorescence intensity of these three kinds of NPs was strongest in liver, followed by the tumor, indicating that the NPs were metabolized via the liver (Figure 4D).

### Evaluation of the *in vivo* Anti-tumor Capacity of PMPNs

**FIGURE 5:**
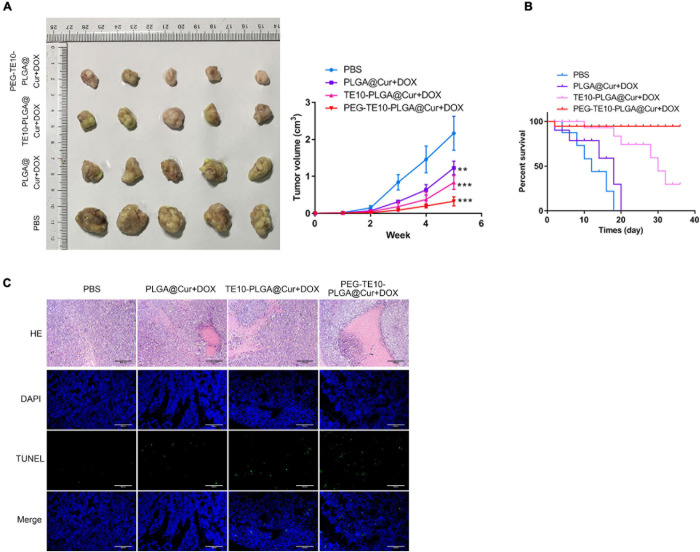
*In vivo* anti-tumor activity of PMPNs. **(A)** Changes of tumor volume during the 16 days treatment of PMPNs. **(B)** The survival percent of mice treated with saline, PLGA@Cur + DOX, TE10-PLGA@Cur + DOX and PEG-TE10-PLGA@Cur + DOX during 40 days. **(C)** H&E and TUNEL staining of tumor tissue section of mice in the four groups. **P < 0.01, ***P < 0.001 vs. control group.

We attempted to explore the anti-tumor capacity of the synthesized PMPNs *in vivo*, to this end, we xenografted TE10/DOX cells to nude mice. PLGA@Cur + DOX or TE10-PLGA@Cur + DOX or PEG-TE10-PLGA@Cur + DOX was injected into each group of mice after tumor formation. Changes in the tumor volume were measured and compared (Figure 5A). The tumor growth rate of mice treated with PEG-TE10-PLGA@Cur + DOX or TE10-PLGA@Cur + DOX was prolonged compared with the control group (*P* < 0.01). The effect of PEG-TE10@PLGA@Cur + DOX treatment was slightly better than that of TE10@PLGA@Cur + DOX, indicating that the modification with PEG increased the circulation time of NPs in the blood and prevented them from being cleaned up, thus the encapsulated drug could be released continuously and killed the tumor. The therapeutic effect of the PLGA@Cur + DOX group was only better than that of the control group (*P* < 0.05), indicating that although PLGA could sustained-released the drug, it is quickly eliminated by immune system due to the absence of targeting of homologous cell membrane and stealth of PEG. The monitoring of the overall survival of mice in each group was consistent with the above results (Figure 5B). The tumor tissue sections of each group of mice were stained with H&E and TUNEL to evaluate necrosis and apoptosis. Figure 5C showed that the tumor cells in mice treated with PEG-TE10-PLGA@Cur + DOX had the most severe necrosis and apoptosis, and the most significant antitumor effect in these four groups.

### Biosafety Assessment of PMPNs

**FIGURE 6:**
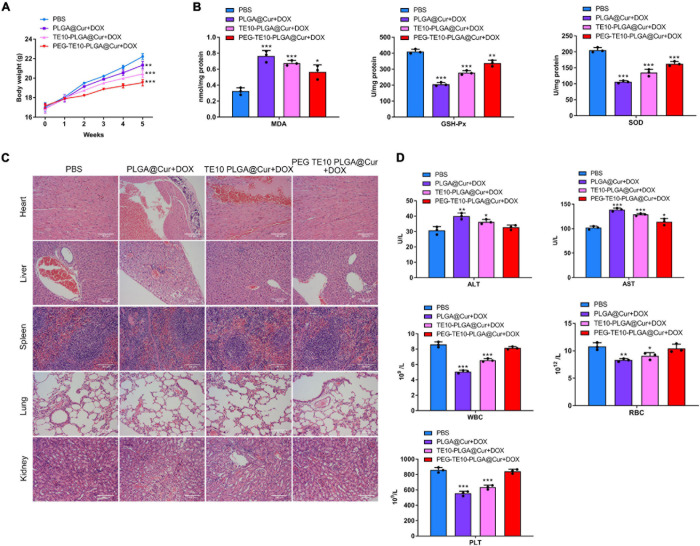
System safety assessment. **(A)** Body weight changes of mice treated with saline, PLGA@Cur + DOX, TE10-PLGA@Cur + DOX and PEG-TE10-PLGA@Cur + DOX. **(B)** Detection of the oxidative stress in mouse cardiomyocytes. **(C)** H&E staining of five major tissue sections of mice. **(D)** Detection of the concentration of ALT, AST, WBC, RBC, and PLT in the serum of mice. *P < 0.05, **P < 0.01, ***P < 0.001 vs. control group.

It has been confirmed that the synthesized PMPNs have certain anti-tumor effect *in vivo* and *in vitro*, and we want to further investigate its biosafety in normal organs and tissues. First, we assessed the weight changes of mice after xenograft and treatment with the PMPNs. The weight gain of mice treated with PEG-TE10-PLGA@Cur + DOX was much higher than that of PLGA@Cur + DOX group (*P* < 0.01) (Figure 6A). Then, we evaluated the oxidative stress level of the cardiomyocytes of the mice in each group. The concentrations of MDA, GSH-Px and SOD in the cardiomyocytes of the mice treated with PEG-TE10-PLGA@Cur + DOX were almost the same as those of the control group, indicating that the treatment of PEG-TE10-PLGA@Cur + DOX did not cause oxidative stress damage to the cardiomyocytes (Figure 6B). Next, we performed H&E staining on the main organs of the mice. There was little tissue damage in the PEG-TE10-PLGA@Cur + DOX and TE10-PLGA@Cur + DOX treatment group, while the heart, liver and kidney of mice in the PLGA@Cur + DOX group showed a certain degree of damage (Figure 6C). Moreover, the biochemical analysis of serum showed that ALT, AST, WBC, RBC, and PLT levels of the mice in the PEG-TE10-PLGA@Cur + DOX treatment group had no significant difference compared with the saline group, while the levels of ALT and AST in the serum of mice in the PLGA@Cur + DOX and TE10-PLGA@Cur + DOX treatment group increased, and the levels of WBC, RBC and PLT decreased significantly (Figure 6D). The above results implied that PEG-TE10-PLGA@Cur + DOX had high biological safety and almost no damage to normal tissues and organs.

## Discussion

DOX is a broad-spectrum anti-tumor drug that can be used to treat various cancers, including esophageal carcinoma, hepatocellular carcinoma, and ovarian carcinoma (Rivankar, 2014). However, its clinical application is restricted by side effects such as cardiotoxicity and susceptible to drug resistance (Chen et al., 2018). Cur is a natural active substance extracted from traditional Chinese medicine Turmeric. Studies have shown that Cur has many functions, such as anti-inflammatory, anti-oxidant, and anti-fibrosis (Kocaadam and Şanlier, 2017). Besides, Cur has also been found to have anti-tumor properties. Liu et al. (2018) found that Cur induced the apoptosis of esophageal squamous cell carcinoma by inhibiting the phosphorylation of STAT3 and blocking its downstream signal pathway. Xiang et al. (2020) pointed out that Cur inhibited human colorectal cancer. They considered that Cur exerted anti-tumor effect through regulating the NF-κB pathway, thus inhibiting the metastasis of tumor cells and promoting the apoptosis of colorectal cancer cells. In addition, studies have found that Cur regulates the level of ROS in tumors, reverses tumor MDR, and reduces the side effects of chemotherapeutic drugs (Larasati et al., 2018; Momtazi-Borojeni et al., 2019). Cur can be used as a sensitizer for a variety of chemotherapeutic drugs including DOX, which can significantly improve the therapeutic effect (Batra et al., 2019). Although Cur has many advantages, it still has the characteristics of poor water solubility, fast clearance and high clearance rate *in vivo*, which limit its clinical application (Anand et al., 2007). For the sake of overcoming the deficiency of DOX and Cur, we adopted the biodegradable nano-carrier PLGA to co-load DOX and Cur, which can not only sustained drug release, but also improve the bioavailability of the drugs and maximize the advantages of the combination of DOX and Cur. In this paper, we found that DOX and CUR had a significant synergistic therapeutic effect, and the tumor killing effect on MDR esophageal carcinoma was significantly better than using DOX alone.

In recent years, the research on biomimetic nanomedicine using cell membrane has become a hot topic. Hu et al. (2011) modified PLGA with erythrocyte membranes to achieve long-cycle characteristics. This independent original research provides a unique method to functionalize nanoparticles. The cell membrane wrapping technology makes full use of the natural characteristics of cell membrane and the principle of bionics. It transfers the cell membrane and various surface molecules carried by the membrane to the encapsulated NPs and endows them with different biological functions. NPs wrapped by tumor cell membranes have active targeting effects compared to NPs wrapped by other cell membranes. In addition to EPR effect, tumor cell membrane-coated NPs can also actively aggregate to the target sites using homologous recognition mechanism. Chen et al. (2016) loaded the photosensitizer indocyanine green (ICG) with PLGA and coated it with the cell membrane of MCF-7 cells to prepare the ICNP with homologous targeting function. *In vivo* and *in vitro* experiments have confirmed that ICNP can target human breast cancer cells for imaging and photothermal therapy. Liu et al. (2019) coated MCF-7 cell membrane on copper/manganese silicate nanospheres (CMSN), which entered tumor cells through homologous targeting, and killed tumor cells through the synergy of photodynamic and chemodynamic therapy. In this manuscript, we used the cell membrane of TE10 to coat the drug-loaded PLGA NPs, thus enabling the drug carrier to have homologous targeting function, and significantly increasing the drug concentration at the target site. In addition, we also modified the cell membrane with PEG, which could not only further extend the circulation time of the drug carrier in the blood, but also reduce the non-specific binding between NPs and serum proteins. Besides, PEGylated modification prevents NPs from being eliminated by the immune system (Ochyl et al., 2018). In this study, we also found that the systemic toxicity of the nanomedicine was significantly reduced after PEG modification (Figure 6), which may be related to the charge blocking effect of PEG layer on NPs. In the process of blood circulation, the surface charge of NPs may cause the aggregation or hemolysis of red blood cells, thus causing systemic toxicity. In addition, the release of hemoglobin and cell debris caused by hemolysis will adsorb to the surface of NPs, which makes NPs easily recognized and cleared by immune cells.

In summary, we synthesized PMPNs for tumor targeting and efficient loading of anti-cancer drugs DOX and Cur. By establishing TE10/DOX cell line, a drug-resistant cell model of esophageal carcinoma, we confirmed that PMPNs could be specifically taken up by TE10/DOX cells, and exhibited good anti-tumor effect *in vitro*. In addition, we also used TE10/DOX to xenograft mice to study the targeting and anti-tumor activity of PMPNs to TE10/DOX *in vivo*. The results showed that PMPNs had excellent targeting and therapeutic effect on TE10/DOX *in vivo*. At the same time, PMPNs had good biological safety, which effectively avoid the side effects of chemotherapy drugs. Our research provides a new strategy for nanomedicine treatment of multidrug resistant esophageal cancer.

## Data Availability Statement

The raw data supporting the conclusions of this article will be made available by the authors, without undue reservation.

## Ethics Statement

The animal study was reviewed and approved by the Ethics Committee of Jiangyin People’s Hospital, the Jiangyin Clinical College of Xuzhou Medical University.

## Author Contributions

YG designed and developed the study. YZ, XX, FW, WS, XL, JZ, BL, and YW performed the experiments. YG and PL analyzed the data and wrote the manuscript. All authors contributed to the article and approved the submitted version.

## Conflict of Interest

The authors declare that the research was conducted in the absence of any commercial or financial relationships that could be construed as a potential conflict of interest.

## Publisher’s Note

All claims expressed in this article are solely those of the authors and do not necessarily represent those of their affiliated organizations, or those of the publisher, the editors and the reviewers. Any product that may be evaluated in this article, or claim that may be made by its manufacturer, is not guaranteed or endorsed by the publisher.
