# Supplementary material for: Surface PEGylated Cancer Cell Membrane-Coated Nanoparticles for Codelivery of Curcumin and Doxorubicin for the Treatment of Multidrug Resistant Esophageal Carcinoma
Source: Front Cell Dev Biol. 2021 Jul 27;9:688070. doi: 10.3389/fcell.2021.688070 (PMC8353447; doi:10.3389/fcell.2021.688070)
Supplement: Supplementary file 1 [file Table_1.docx]

**
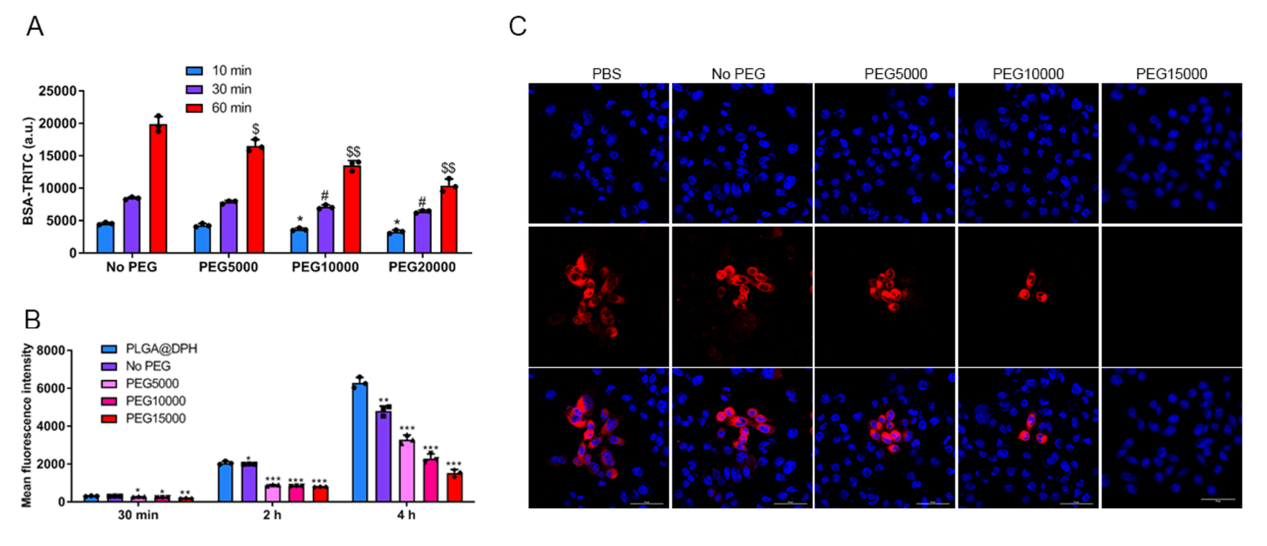
**

**Figure S1. Effects of PEG Surface modification on NPs**. (A) The adsorption of BSA by NPs modified with different molecular weights of PEG. Flow cytometry profile (B) and laser scanning confocal microscope image (C) of the uptake of different molecular weights PEG-modified NPs by macrophages.

**
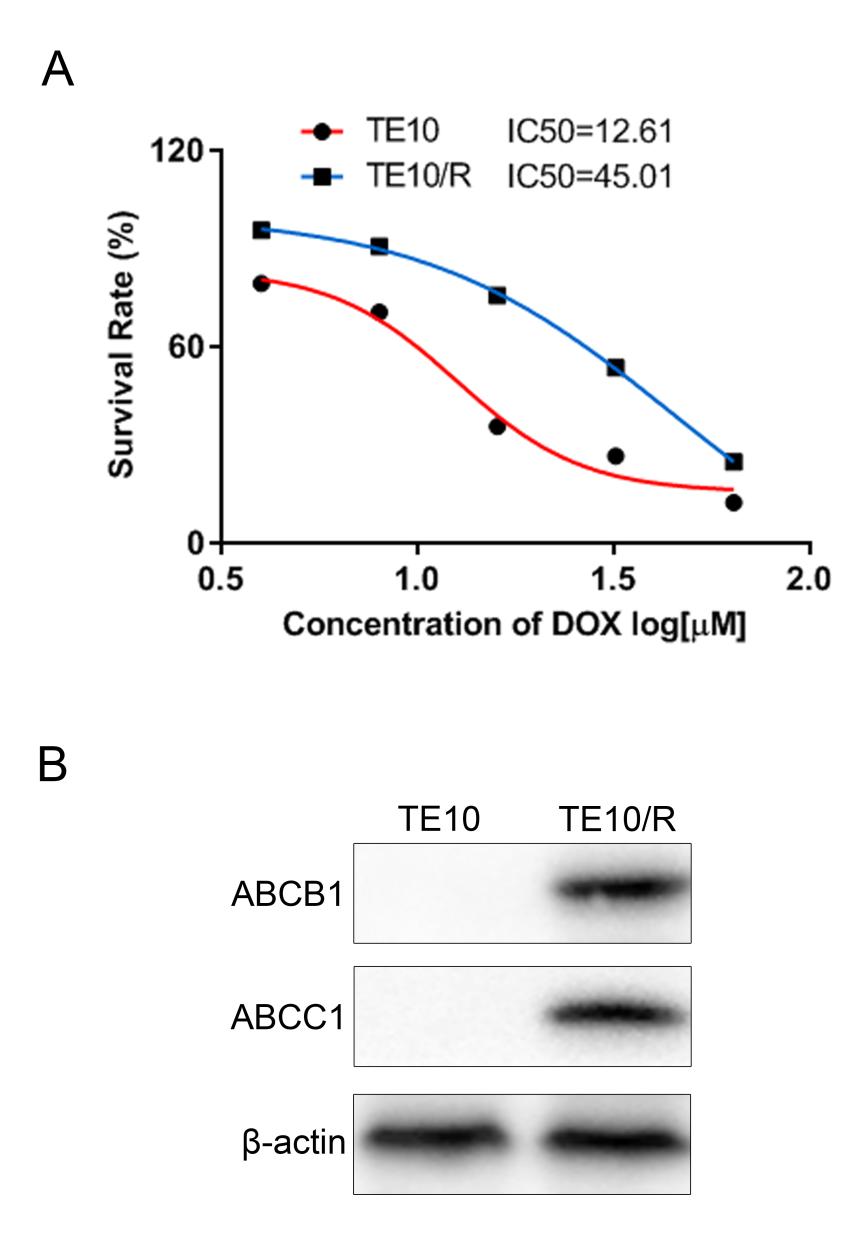
**

**Figure S2. Identification of the DOX-resistant TE10 cells.** (A) *IC_50_* value of TE10 cells and TE10/DOX cells were assessed by CCK-8 assay. (B) Western blot was performed to evaluate the expression level of resistance proteins ABCB1 and ABCC1.

**
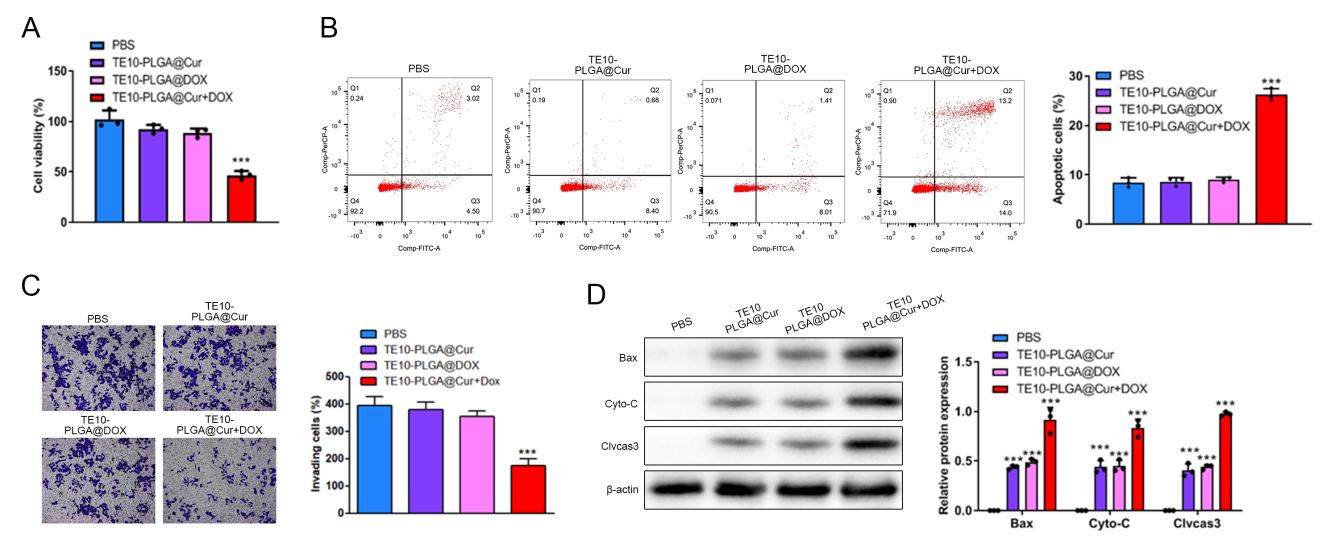
**

**Figure S3. Synergistic effect of DOX and Cur**. CCK-8 analysis (A), flow cytometry (B) and Transwell analysis (C) were conducted to evaluate the synergistic effect of DOX and Cur in the treatment of tumor. (D) The expression of apoptosis-related proteins in TE10/DOX cells was analyzed by Western blot.*** *P* < 0.01.

**
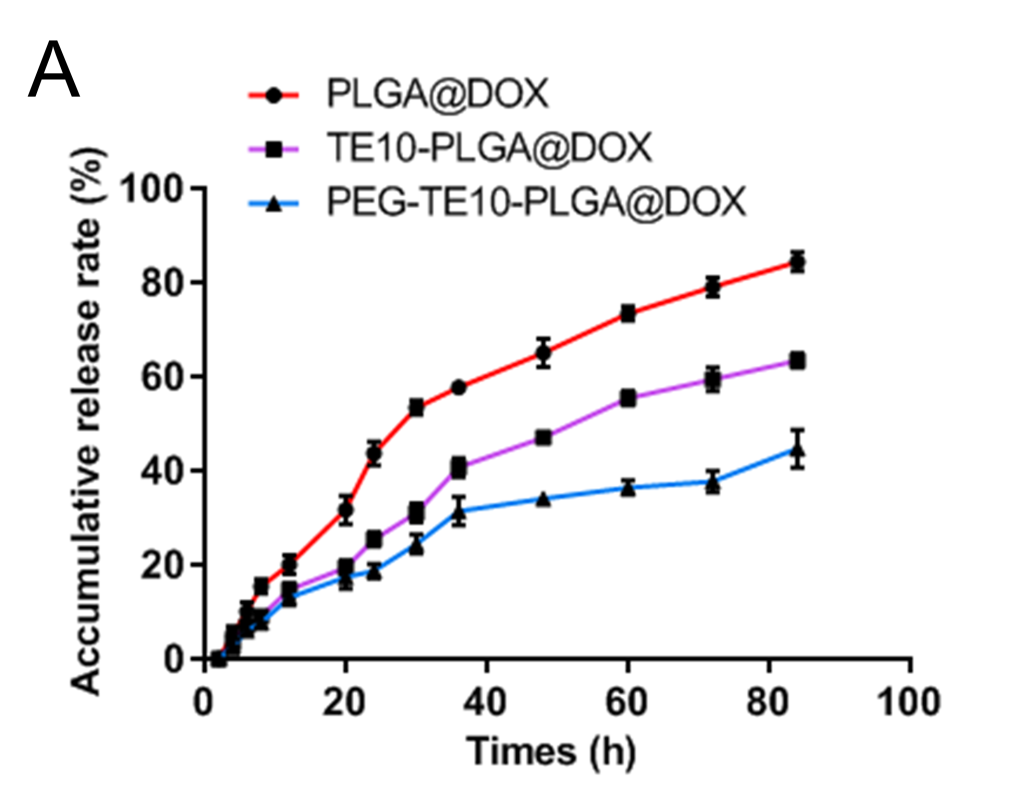
**

**Figure S4. Drug release curve of the NPs**. Fluorescence of DOX at 2, 4, 6, 8, 12, 20, 24, 30, 36, 48, 60, 72, and 84 h after treatment was measured to determine drug release capacity of NPs.
